# Prevalence, reasons, and timing of decisions to withhold/withdraw life-sustaining therapy for out-of-hospital cardiac arrest patients with extracorporeal cardiopulmonary resuscitation

**DOI:** 10.1186/s13054-023-04534-2

**Published:** 2023-06-27

**Authors:** Hiromichi Naito, Masaaki Sakuraya, Takashi Hongo, Hiroaki Takada, Tetsuya Yumoto, Takashi Yorifuji, Toru Hifumi, Akihiko Inoue, Tetsuya Sakamoto, Yasuhiro Kuroda, Atsunori Nakao, SAVE-J II Study Group

**Affiliations:** 1 Department of Emergency, Critical Care, and Disaster Medicine, Okayama University Faculty of Medicine, Graduate School of Medicine, Dentistry, and Pharmaceutical Sciences, 2-5-1 Shikata, Kita, Okayama 700-8558 Japan; 2 Department of Emergency and Intensive Care Medicine, JA Hiroshima General Hospital, 1-3-3 Jigozen, Hatsukaichi, Hiroshima 738-0042 Japan; 3 Department of Critical Care Medicine and Trauma, National Hospital Organization Disaster Medical Center, 3256 Midori, Tachikawa, Tokyo 190-0014 Japan; 4 Department of Epidemiology, Okayama University Faculty of Medicine, Dentistry, and Pharmaceutical Science, 2-5-1 Shikata, Kita, Okayama 700-8558 Japan; 5 Department of Emergency and Critical Care Medicine, St. Luke’s International Hospital, Akashi, Chuo, Tokyo 104-8560 Japan; 6 Department of Emergency and Critical Care Medicine, Hyogo Emergency Medical Center, 1-3-1 Wakihamakaigandori, Chuo, Kobe, Hyogo 651-0073 Japan; 7 Department of Emergency Medicine, Teikyo University School of Medicine, 2-11-1 Kaga, Itabashi, Tokyo 173-8606 Japan; 8 Department of Emergency, Disaster, and Critical Care Medicine, Faculty of Medicine, Kagawa University, 1750-1 Ikenobe, Miki, Kita, Kagawa 761-0793 Japan

**Keywords:** Clinical decision-making, Treatment limitation, Futility, Post-cardiac arrest syndrome, ECPR

## Abstract

**Background:**

Extracorporeal cardiopulmonary resuscitation (ECPR) is rapidly becoming a common treatment strategy for patients with refractory cardiac arrest. Despite its benefits, ECPR raises a variety of ethical concerns when the treatment is discontinued. There is little information about the decision to withhold/withdraw life-sustaining therapy (WLST) for out-of-hospital cardiac arrest (OHCA) patients after ECPR.

**Methods:**

We conducted a secondary analysis of data from the SAVE-J II study, a retrospective, multicenter study of ECPR in Japan. Adult patients who underwent ECPR for OHCA with medical causes were included. The prevalence, reasons, and timing of WLST decisions were recorded. Outcomes of patients with or without WLST decisions were compared. Further, factors associated with WLST decisions were examined.

**Results:**

We included 1660 patients in the analysis; 510 (30.7%) had WLST decisions. The number of WLST decisions was the highest on the first day and WSLT decisions were made a median of two days after ICU admission. Reasons for WLST were perceived unfavorable neurological prognosis (300/510 [58.8%]), perceived unfavorable cardiac/pulmonary prognosis (105/510 [20.5%]), inability to maintain extracorporeal cardiopulmonary support (71/510 [13.9%]), complications (10/510 [1.9%]), exacerbation of comorbidity before cardiac arrest (7/510 [1.3%]), and others. Patients with WLST had lower 30-day survival (WLST vs. no-WLST: 36/506 [7.1%] vs. 386/1140 [33.8%], *p* < 0.001). Primary cerebral disorders as cause of cardiac arrest and higher severity of illness at intensive care unit admission were associated with WLST decisions.

**Conclusion:**

For approximately one-third of ECPR/OHCA patients, WLST was decided during admission, mainly because of perceived unfavorable neurological prognoses. Decisions and neurological assessments for ECPR/OHCA patients need further analysis.

**Supplementary Information:**

The online version contains supplementary material available at 10.1186/s13054-023-04534-2.

## Background

As an advanced treatment strategy, extracorporeal cardiopulmonary resuscitation (ECPR) continues to improve the care of patients after out-of-hospital cardiac arrest (OHCA) [1–3]. Despite its benefits, ECPR is still associated with significant morbidity [4], including brain ischemia/injury [5] leading to prolonged neurological impairment. Survival with good neurological outcome after ECPR is still not sufficiently high [6]. The decision to initiate ECPR must be made under emergency conditions with time constraints and uncertainty of prognosis. After ECPR induction, it may become apparent that the patient’s predicted prognosis is extremely poor and aggressive treatment is no longer reasonable. As a result, it may become necessary to adjust goals for end-of-life decision-making, including establishing treatment limits or discontinuing extracorporeal membrane oxygenation (ECMO) or life-sustaining therapies.

Discontinuing ECPR or other life-sustaining therapies raises a variety of ethical concerns due to the lack of internationally accepted guidelines for termination of resuscitation [7], uncertainty of neurological prognosis testing in the acute phase [8], and the medical futility of continuing ECPR in certain situations [9]. Although treatment may sometimes be withheld or discontinued for cases with extremely poor prognoses, there is little information concerning decision criteria to withhold/withdraw life-sustaining therapy (WLST) for OHCA patients after ECPR. Past studies have been limited by sample size [10] or by including only patients who died after the decision to withdraw life-sustaining therapy [11].

The aim of this study was to describe WLST decisions for OHCA patients after ECPR. Prevalence, reasons, and timing for WLST decisions were examined. Further, the differences between ECPR/OHCA patients with or without WLST were compared.

## Methods

### Study design

This was a secondary analysis of a nationwide, retrospective, observational ECPR study (SAVE-J II). The Okayama University Graduate School of Medicine, Dentistry, and Pharmaceutical Sciences and the Okayama University Hospital Ethics Committee approved the study (K2209-018) and waived the requirement for written informed consent.

### Data collection

Data for the analysis was obtained from the SAVE-J II study (ECPR data collected from 36 intensive care units (ICUs) in Japan from 2013 to 2018). The study design and data collection methods of the SAVE-J II study have been described previously [12]. The SAVE-J II study included consecutive patients with OHCA aged ≥ 18 years old who were resuscitated with ECPR at participating institutions during the study period.

### ECPR and ECMO care in the ICU

In Japan, acute care physicians or emergency physician intensivists generally direct the entire clinical practice of OHCA, including ECPR; they provide the continuum of care from the emergency department to post-resuscitation or ECMO care in the ICU. In addition, patient management commonly involves collaboration with cardiologists [13, 14].

### Patient selection and endpoint

Patients registered in the SAVE-J II study were screened for eligibility. Patients treated with ECPR for OHCA with medical causes were included [15], and patients with missing WLST data were excluded. The primary outcome of the study was WLST decision in OHCA patients with ECPR. Prevalence, reasons, and timing for WLST decisions were described. The secondary outcome of the study was 30-day survival, 30-day neurological outcomes, and medical cost during hospitalization. Differences between ECPR/OHCA patients with or without WLST were compared.

### Definitions and measurements

WLST was defined as medical interventions withheld or withdrawn, including ECMO or other life-sustaining treatments, with the understanding that the patient would most likely die from the underlying post-cardiac arrest syndrome or other causes. In this study, WLST was the combination of two categories: withholding life-sustaining treatment and withdrawal of life-sustaining treatment. Withholding life-sustaining treatment is commonly considered the establishment of treatment limits with no escalation of use of devices (including ECMO, ventilator, etc.) or drugs; withdrawal of life-sustaining treatment in Japan is commonly considered the termination of device use, pharmacological interventions, and/or other therapies. After a thorough review of each patient’s medical record, the physician in charge at each study site retrospectively determined the reason for the WLST decision and the date when the decision was made. One of the following six reasons were determined for each case: (1) inability to maintain extracorporeal cardiopulmonary support; (2) complications; (3) perceived unfavorable neurological prognosis; (4) perceived unfavorable cardiac/pulmonary prognosis; (5) exacerbation of comorbidity before cardiac arrest; (6) or others. The WLST group was defined as patients with WLST decisions (combination of withholding and withdrawal of life-sustaining treatment) during their stay in the participating hospital, and the no WLST group was defined as patients with no WLST.

The following patient data were collected from the SAVE-J II study database [12]: gender, age, cardiac arrest location, Eastern Cooperative Oncology Group performance status (PS) before admission (a lower PS score indicates a higher level of performance of daily activities: a score of 0 indicates that the patient is fully active and able to carry out all activities without restriction, while a score of 4 indicates that the patient is completely disabled and bedridden [16]), initial cardiac rhythm, presumed cardiac origin, witness of cardiac arrest, bystander cardiopulmonary resuscitation (CPR), ECPR information (time from emergency medical services scene arrival to ECMO pump on, use of intra-aortic balloon pump, ECMO-related complications), and ICU data/treatments (pH, serum lactate level, sequential organ failure assessment [SOFA] score, pupil diameter at ICU admission, and comorbidity/treatments in the ICU). In this study, SOFA score was defined without the respiratory score due to the uncertainty of effects of ECMO and mechanical ventilation on the respiratory score (i.e., the max score is 16 points). WLST data, including the time the decision was obtained and the reason for WLST, was collected and recorded. Favorable neurological outcomes, defined as Cerebral Performance Category (CPC) score of 1 or 2 at 30 days after cardiac arrest, were obtained. Survival at 30 days and medical cost incurred during the hospitalization were recorded.

### Statistical analysis

Continuous variables were described using median with interquartile ranges. Categorical variables are described using percentages. Comparisons between two groups were made using the Mann–Whitney U test for continuous variables and the chi-square test for categorical variables. Kaplan–Meier survival curve was drawn for the comparison of 30-day survival of the WLST and no WLST groups with log-rank test. A multiple logistic regression model was applied to estimate adjusted odds ratios (ORs) with their 95% confidence intervals for the primary outcome. We selected confounding variables as follows: age, gender, PS before cardiac arrest, primary cerebral disorders as cause of cardiac arrest, time from scene to ECMO pump on, pupil diameter (≥ 6 mm) on ICU admission, SOFA score at ICU admission, lactate level at ICU admission, and ECMO complications. These variables were selected based on previous reports and our hypothesis was that these variables were potentially associated with WLST [10, 11]. All statistical analysis was performed using Stata version 17 (StataCorp LP, College Station, TX).

## Results

### Patient characteristics

**Fig. 1:**
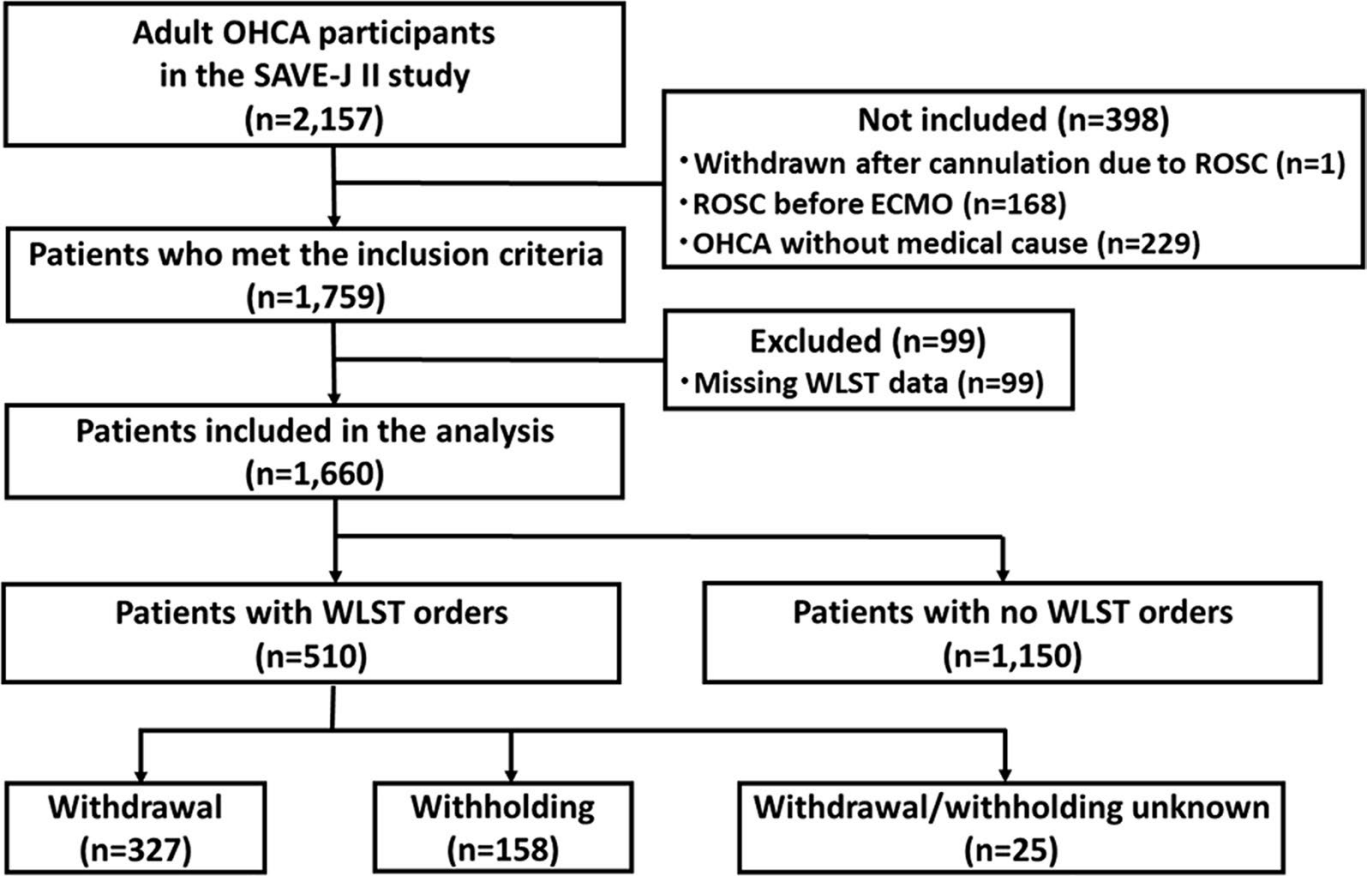
Flow diagram of patients analyzed. *ECMO* Extracorporeal membrane oxygenation, *ECPR* Extracorporeal cardiopulmonary resuscitation, *OHCA* Out-of-hospital cardiac arrest, *ROSC* Return of spontaneous circulation, *WLST* Withholding/withdrawal of life-sustaining therapy

Figure 1 shows a flow chart depicting how patients were determined to be eligible for analysis. During the study period, data on 2157 patients were entered into the SAVE-J II Study registry. A total of 1759 patients met the inclusion criteria. Patients meeting the exclusion criteria (99 patients missing WLST data) were excluded. Finally, 1660 patients were included in the analysis, 510 patients (30.7%) of which had WLST decisions during admission. Of the 510 WLST patients, the decision was made to withdraw life-sustaining treatment for 327 patients and the decision was made to withhold life-sustaining treatment was made for 158 patients.

Patient characteristics are shown in Table 1. In the univariable analysis, WLST patient age was higher (WLST vs. no WLST: 62 [51–70] vs. 61 [49–69], *p* = 0.032). Patients with WLST were more likely to have primary cerebral disorders as the cause of cardiac arrest (32/502 [6.3%] vs. 18/1137 [1.5%], *p* < 0.001), higher serum lactate levels at ICU admission (8.3 [5.7–12.0] vs. 7.6 [5.0–11.3] mmol/L, *p* = 0.008), a higher prevalence of dilated pupils (≥ 6 mm) on ICU admission (57/420 [13.5%] vs. 75/813 [9.2%], *p* = 0.019), and a higher prevalence of acute renal failure (218/455 [47.9%] vs. 335/894 [37.4%], *p* < 0.001).

**Table 1:** Characteristics of OHCA patients who underwent ECPR for medical causes

	All (n = 1660)	WLST (n = 510)	no WLST (n = 1150)	*p*-value
*Baseline characteristics*				
Male gender, n (%)	1382/1660 (83.2)	427/510 (83.7)	965/1150 (83.9)	0.279
Age, median [IQR]	61 [50–69]	62 [51–70]	61 [49–68]	0.032
Cardiac arrest location				0.079
Home, n (%)	674/1660 (40.6)	226/510 (44.3)	448/1150 (38.9)	
Public, n (%)	693/1660 (41.7)	193/510 (37.8)	500/1150 (43.4)	
Other, n (%)	293/1660 (17.6)	91/510 (17.8)	202/1150 (17.5)	
PS before admission				0.107
PS 0, n (%)	1462/1621 (90.1)	441/494 (89.2)	1021/1127 (90.5)	
PS 1, n (%)	127/1621 (7.8)	37/494 (7.4)	90/1127 (7.9)	
PS 2, n (%)	21/1621 (1.3)	11/494 (2.2)	10/1127 (0.8)	
PS 3, n (%)	11/1621 (0.6)	5/494 (1.0)	6/1127 (0.5)	
PS 4, n (%)	0/1621 (0)	0/494 (0)	0/1127 (0)	
Initial rhythm				
VF/VT, n (%)	1071/1646 (65.0)	318/503 (63.2)	753/1143 (65.8)	0.297
PEA/asystole, n (%)	575/1646 (34.9)	185/503 (36.7)	390/,1143 (34.1)	0.297
Presumed cardiac origin, n (%)	1358/1648 (82.4)	403/505 (79.8)	955/1143 (83.5)	0.065
Presumed cause of cardiac arrest				
Acute coronary syndrome, n (%)	924/1639 (56.3)	274/502 (54.5)	650/1137 (57.1)	0.330
Arrhythmia, n (%)	232/1639 (14.1)	62/502 (12.3)	170/1137 (14.9)	0.164
Pulmonary embolism, n (%)	52/1639 (3.1)	20/502 (3.9)	32/1137 (2.8)	0.213
Aortic dissection, n (%)	92/1639 (5.6)	18/502 (3.5)	74/1137 (6.5)	0.018
Primary cerebral disorders n (%)	50/1639 (3.0)	32/502 (6.3)	18/1137 (1.5)	< 0.001
Other, n (%)	289/1639 (17.6)	96/502 (19.1)	193/1137 (16.9)	0.031
Witnessed collapse, n (%)	1295/1652 (78.3)	407/506 (80.4)	888/1146 (77.4)	0.180
Bystander CPR, n (%)	949/1633 (58.1)	290/500 (58.0)	659/,1133 (58.1)	0.951
*ECPR*				
Time interval (min), median [IQR]				
EMS arrival-ECMO pump on	41 [28–49]	41 [27–48]	41 [28–49]	0.242
IABP use, n (%)	1018/1656 (61.4)	300/510 (58.8)	718/1146 (62.6)	0.139
ECMO-related complications, n (%)	410/1660 (24.7)	125/510 (24.5)	285/1150 (24.7)	0.905
Procedure‑related complications, n (%)	256/1653 (15.4)	75/505 (14.8)	206/1148 (15.7)	0.653
Brain ischemia, n (%)	6/1660 (0.3)	2/510 (0.3)	4/1150 (0.3)	0.890
Mesenteric ischemia, n (%)	9/1654 (0.5)	3/505 (0.5)	6/1149 (0.5)	0.855
Cerebral hemorrhage, n (%)	16/1644 (0.9)	8/510 (1.5)	8/1150 (0.7)	0.093
Gastrointestinal bleeding, n (%)	35/1654 (2.1)	19/508 (3.7)	16/1146 (1.4)	0.002
Other hemorrhagic complication, n (%)	101/1654 (6.1)	28/508 (5.5)	73/1146 (6.3)	0.829
ECMO mechanical complication, n (%)	27/1628 (1.6)	11/500 (2.2)	16/1128 (1.4)	0.255
*ICU data and treatments*				
Blood gas pH (ICU admission), median [IQR]	7.30 [7.20–7.39]	7.30 [7.19–7.40]	7.30 [7.20–7.38]	0.848
Serum lactate (mmol/L), median [IQR]	7.8 [5.3–11.6]	8.4 [5.7–12.0]	7.6 [5.0–11.3]	0.008
SOFA score, median [IQR]	12 [8–13]	12 [8–13]	12 [9–13]	0.460
Pupil diameter ≥ 4 mm on ICU admission, n (%)	398/1233 (32.2)	138/420 (32.8)	260/813 (31.9)	0.755
Pupil diameter ≥ 6 mm on ICU admission, n (%)	132/1233 (10.7)	57/420 (13.5)	75/813 (9.2)	0.019
Sepsis, n (%)	135/1368 (9.8)	43/457 (9.4)	92/911 (10.1)	0.687
Septic shock, n (%)	87/1363 (6.3)	35/457 (7.6)	52/906 (5.7)	0.171
Acute renal failure, n (%)	553/1349 (40.9)	218/455 (47.9)	335/894 (37.4)	< 0.001
Ventilator-associated pneumonia, n (%)	162/1342 (12.0)	45/449 (10.0)	117/893 (13.1)	0.102
Vasopressor use, n (%)	629/1362 (46.1)	192/454 (42.2)	437/908 (48.1)	0.042
Renal replacement therapy, n (%)	236/1357 (17.3)	69/453 (15.2)	167/904 (18.4)	0.137
Tracheostomy, n (%)	245/1642 (14.9)	52/505 (10.3)	193/1137 (16.9)	< 0.001
Temperature management, n (%)				
32 °C-below 36 °C	828/1112 (74.4)	237/328 (72.2)	591/784 (75.3)	0.276
36 °C or higher	284/1112 (25.5)	91/328 (27.7)	193/784 (24.6)	0.276
Temperature management canceled, n (%)	324/1095 (29.5)	106/322 (32.9)	218/773 (28.2)	0.119

*CPR* Cardiopulmonary resuscitation, *ECMO* Extracorporeal membrane oxygenation, *ECPR* Extracorporeal cardiopulmonary resuscitation, *EMS* Emergency medical service, *IABP* Intra-aortic balloon pump, *ICU* Intensive care unit, *IQR* Interquartile range, *SOFA* Sequential organ failure assessment, *OHCA* Out-of-hospital cardiac arrest, *PEA* Pulseless electrical activity, *PS* Performance status, *VF* Ventricular fibrillation, *VT* Ventricular tachycardia, *WLST* Withholding/withdrawal of life-sustaining therapy

### Status and reasons for WLST decisions, time to obtain WLST decisions

**Fig. 2:**
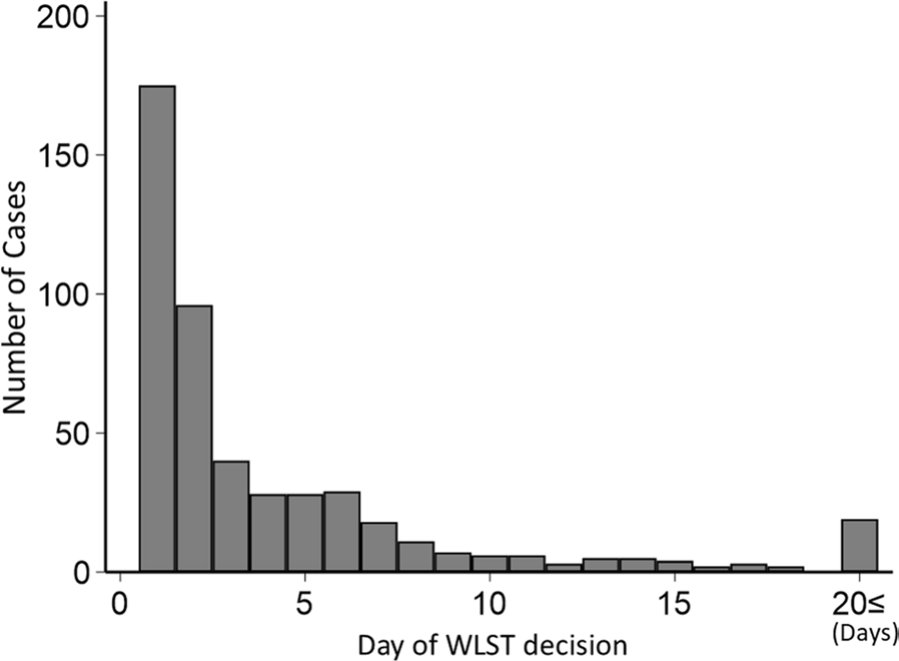
Timing of WLST decisions. The number of WLST decisions was the highest on the first day; the number of WLST decisions decreased with time. *WLST* Withholding/withdrawal of life-sustaining therapy

Table 2 shows the reasons for WLST decisions. Reasons for WLST decisions were perceived unfavorable neurological prognosis (300/510 [58.8%]), perceived unfavorable cardiac/pulmonary prognosis (105/510 [20.5%]), inability to maintain extracorporeal cardiopulmonary support (71/510 [13.9%]), complications (10/510 [1.9%]), exacerbation of comorbidity before cardiac arrest (7/510 [1.0%]), and others (4/510 [1.0%]). Proportions for each WLST reason did not differ between patients with withdrawal of life-sustaining treatment decisions and patients with withholding of life-sustaining treatment decisions. WLST was decided at a median of 2 [1–6] days. Figure 2 shows the days on which WLST decisions were made. The number of WLST decisions was the highest on the first day; the number of WLST decisions decreased with time. The trend for WLST did not differ by WLST reason (Additional file 1).

**Table 2:** Reasons for withdrawal/withholding of life-sustaining treatment

	All WLST (n = 510)	Withholding (n = 327)	Withdrawal (n = 158)	*p*-value
Perceived unfavorable neurological prognosis, n (%)	300/510 (58.8)	198/327 (60.5)	98/156 (62.8)	0.632
Perceived unfavorable cardiac/pulmonary prognosis, n (%)	105/510 (20.5)	69/327 (21.1)	31/156 (19.8)	0.755
Inability to maintain extracorporeal cardiopulmonary support, n (%)	71/510 (13.9)	42/327 (12.8)	24/156 (15.3)	0.447
Complications, n (%)	10/510 (1.9)	8/327 (2.4)	2/156 (1.2)	0.401
Exacerbation of comorbidity before cardiac arrest, n (%)	7/510 (1.3)	6/327 (1.8)	1/156 (0.6)	0.305
Other, n (%)	4/510 (1.0)	4 /327 (1.2)	0/156 (0)	0.165
Unknown, n (%)	13/510 (2.5)	0 (0)	2 (1.2)	

*WLST* Withdrawal/withholding of life-sustaining treatment. Withdrawal or withholding status were unknown in 25 patients

### One-month survival and other patient outcomes

**Fig. 3:**
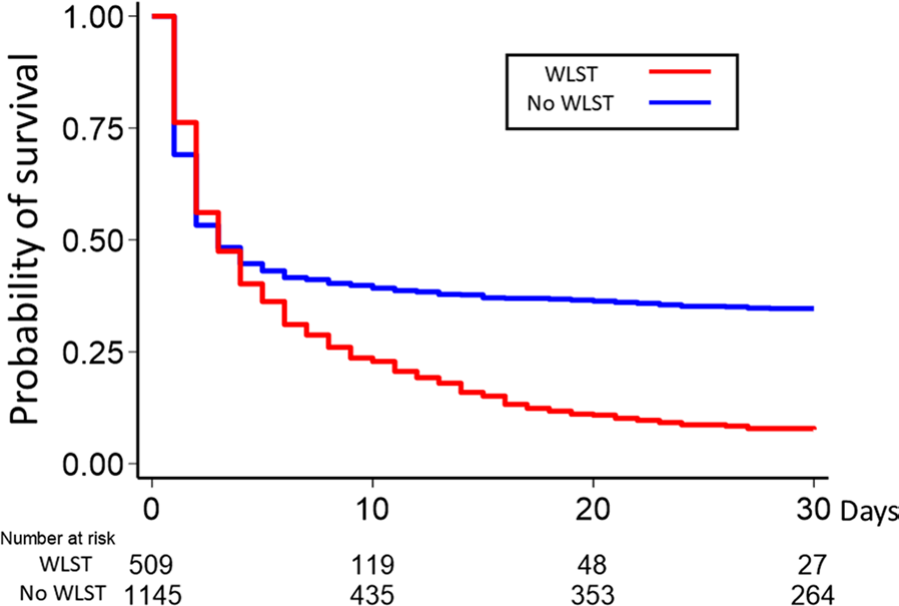
Kaplan–Meier survival curve was drawn for the comparison of 30-day survival of the WLST and no WLST groups with log-rank test. Thirty-day survival was better in the no WLST group (*p* < 0.001). WLST: withholding/withdrawal of life-sustaining therapy

Table 3 shows patient outcomes. Overall, 422/1646 (25.6%) of the patients survived at 30 days. Favorable neurological outcomes were seen in 214/1646 (13.0%) of the patients. A median of $23,000 US dollars was spent to treat the patients during hospitalization. Thirty-day survival (*p* < 0.001, see also Fig. 3) and 30-day neurological outcomes were better in the no WLST group (*p* < 0.001), while medical cost during hospitalization was lower in the WLST group (*p* < 0.001). Thirty-day survival was better in the withholding life-sustaining treatment decision group compared to the withdrawal of life-sustaining treatment decision group (*p* < 0.001, Additional file 2).

**Table 3:** Patient survival, neurological outcomes, and medical cost

	All (n = 1660)	WLST (n = 510)	No WLST (n = 1150)	*p*-value
Survival at 30 days, n (%)	422/1646 (25.6)	36/506 (7.1)	386/1140 (33.8)	< 0.001
Favorable neurological outcome at 30 days, n (%)^a^	214/1646 (13.0)	0/506 (0)	214/1140 (18.7)	< 0.001
Medical cost during hospitalization ($), median [IQR]^b^	23,000 [10,000–38,000]	21,000 [10,000–31,000]	24,000 [11,000–43,000]	< 0.001

*WLST* withholding/withdrawal of life-sustaining therapy. ^a^Favorable neurological outcome was defined as Cerebral Performance Category score of 1 or 2. ^b^Medical cost was converted from Yen to US Dollars (1$ = 100 Yen). Survival data missing for 14 patients. Neurological outcome data missing for 14 patients. Medical cost during hospitalization missing for 89 patients

### Factors related to WLST decisions

Table 4 shows the results of multivariable logistic regression for WLST decisions. Primary cerebral disorders as cause of cardiac arrest (adjusted OR 6.67 [1.29–34.5], *p* = 0.023), SOFA score on ICU admission (adjusted OR 1.05 [1.00–1.09], *p* = 0.017), and lactate level on ICU admission (adjusted OR 1.04 [1.01–1.08], *p* = 0.007) were the factors associated with WLST decisions after adjustment.

**Table 4:** Factors related to WLST decisions

	Adjusted OR (95% CI)	*p*-value
Age	1.00 (0.99–1.02)	0.084
Gender male	1.00 (0.67–1.49)	0.986
PS before cardiac arrest	1.03 (0.70–1.52)	0.862
Primary cerebral disorders	6.67 (1.29–34.5)	0.023
Time to ECMO (EMS arrival to ECMO pump-on)	0.99 (0.98–1.00)	0.577
Pupil diameter (≥ 6 mm) on ICU admission	1.29 (0.79–2.09)	0.300
SOFA score on ICU admission	1.05 (1.00–1.09)	0.017
Lactate level on ICU admission	1.04 (1.01–1.08)	0.007
Any ECMO complication	1.17 (0.85–1.61)	0.323

Adjusted for each variable

*CI* Confidence interval, *ECMO* Extracorporeal membrane oxygenation, *EMS* Emergency medical services, *ICU* Intensive care unit, *SOFA* Sequential organ failure assessment, *OR* Odds ratio, *PS* Performance status, *WLST* Withdrawal/withholding of life sustaining therapy

## Discussion

In this study, we described the prevalence and reasons for WLST decisions in patients who underwent ECPR in a Japanese nationwide registry. WLST decisions were made most frequently on the first day and the median time until WLST decisions were made was two days in our ECPR population. Surprisingly, the majority of attending physicians disclosed that the reason for WLST was perceived unfavorable neurological prognosis, although we could not examine whether they could actually estimate the patient’s prognosis at this early time. Patients experiencing WLST had higher mortalities and incurred lower medical costs compared to those without WLST.

Approximately one third (30.7%) of our ECPR population had WLST decisions during their hospital stay. A previous report demonstrated that in a general population of OHCA patients with conventional CPR, WLST at any time/for any reason occurred in 43% of the patients [17]. Similarly, in the other conventional CPR population study, withdrawal of life-sustaining therapy for perceived poor neurologic prognosis occurred in 25% of hospitalized patients [18]. Although our ECPR cohort had a high proportion (13.0%) of favorable neurological outcomes, the prevalence of WLST was comparable with that of the general conventional CPR OHCA population.

Although guidelines recommend avoiding early WLST in patients after cardiac arrest [19, 20], the timing for WLST decisions was surprisingly early in our ECPR cohort, with a mode of one day and a median of two days after ICU admission, which is different from the conventional CPR population, in which 17% of hospitalized OHCA patients had WLST within three calendar days [17]. Past literature on WLST in ECPR patients is limited; however, similar to our study, the prevalence of early WLST in ECPR patients in these previous studies seems to be high. Our results were consistent with those from a past report that used data from The Extracorporeal Life Support Organization (ELSO) registry. Carlson et al. reported that decisions to withdraw life-sustaining therapy most commonly occurred on day one [11]. Additionally, Haas et al. reported that more than one third of non-survivors’ ECPR was discontinued within 24 h of ECPR initiation, although the number of WLST decisions was not described [21]. Early WLST may eliminate the chance of survival during a time when prognostic estimation is not possible; this may lead to excess mortality. Elmer et al. reported that WLST decisions due to presumed poor neurological prognosis were made within 72 h in one-third of OHCA patients that died in-hospital. If life-sustaining therapy was not withdrawn before 72 h, two prognostic models derived from a large database in North America predicted that 26% of those who died due to early withdrawal of life-sustaining therapy decision might have survived and 16% of those who died might have had functionally favorable survival [22].

In our study, some of the patient’s baseline demographics were associated with WLST. When adjusted in the multivariable regression model, primary cerebral disorders as cause of cardiac arrest, SOFA score on ICU admission, and lactate level on ICU admission were factors associated with WLST. This finding is consistent with that from a previous study showing that severity of illness was associated with WLST within 72 h in ECPR patients [10]. We additionally found that primary cerebral disorders, commonly associated with poor favorable outcomes, were strongly associated with WLST.

Carlson et al. described clinical outcomes of WLST in ECMO patients in a single center; all 73 patients (100%) with WLST decisions died [10]. In our analysis, 7.1% of the WLST group survived until 30 days, but none survived with 30-day good neurological outcomes. We found a significant difference in survival between the WLST group and the no WLST group; further, there were significant differences in survival between patients with withdrawal of life-sustaining therapy and patients with withholding of life-sustaining therapy. These findings raise concerns for early WLST based on presumed unfavorable neurological outcome. Inappropriately predicting a poor neurological outcome may result in a self-fulfilling prophecy. Using such a prognostication to guide early WLST may have a critical impact on survival.

There still are numerous barriers to ECPR implementation; some of the issues with ECPR are medical cost and clinical effectiveness for OHCA patients [23]. Japan's universal coverage charges patients a very low cost, covering most hospitalization costs under national health care expenditures [24]. Still, in this study, medical cost in the WLST group was lower due to early death with WLST. However, our data are insufficient to quantify the social or economic costs for death associated with WLST.

The decision for WLST depends on a variety of factors. Besides the patient's medical condition or medical cost, patient/family cultural and religious beliefs, values, and preferences may affect the decisions [25]. Despite Japanese guidelines regarding end-of-life-care that provide a basis for WLST decisions and decision-making using a multidisciplinary approach, physicians in Japan often prefer to not withhold or withdraw life-sustaining therapy in the ICU [26]. However, this would not apply to specific situations in which neurological damage is so catastrophic that functional recovery is extremely unlikely. Remarkably, a recent study from Japan demonstrated that almost all WLST decisions were made through discussions between patients’ family members and attending emergency physician intensivists/neurosurgeons within 24 h after admission for severe traumatic brain injury [27]. Early WLST decision in our study may be in part attributed to the physician’s perception of futility that the patient would have a poor neurological prognosis or a “bridge to nowhere.” It is also speculated that attending physicians/families decided WLST with the self-fulfilling prophecy that the patient would have an unfavorable outcome. Physicians should be aware that the self-fulfilling prophecy that may result from inappropriate pessimistic neurological prognostication has a detrimental impact on patient outcomes. Withdrawal of life-sustaining therapy is a critical factor for estimated excessive mortality in cardiac arrest with conventional CPR [17, 22] or intracerebral hemorrhage [28]. Future research should aim to coherently clarify the decision-making process for why ECMO care, which is expensive and time-consuming, was withdrawn or withheld as early as 1–2 days in ECPR patients.

### Limitations

The study has several limitations. First, this study was conducted as a post-hoc analysis of data from a Japanese nationwide registry [12]; decisions and treatments of ECPR patients were left to the discretion of attending physicians and family members. We did not have any specific protocol for WLST decisions. We were unable to investigate specific findings that may have underpinned physicians’ perceptions of unfavorable neurological or cardiac/pulmonary prognoses. We did not have data on the WLST decision-making process, so we did not know whether a multidisciplinary team approach was used or whether a family member initiated the decision, or the roles of family members, physicians (emergency physician intensivists, cardiologists, etc.), and other healthcare professionals, including social workers or ethical consultants. Second, the most appropriate reason for WLST was chosen by the researchers of the participating facility based on medical records. Retrospectively choosing one of six possible reasons for WLST is not likely to reflect the complexity of such decisions that also include multiple other factors, including family/attending physician discretion, late found advance directives, or economic factors. Our study did not set specific guidelines for choosing WLST reasons. Finally, this study is based on data from a single nation; culture, ethics, and economic setting may affect WLST decisions, which may limit the generalizability of our findings. However, our data is consistent with previous international registry data [11].

Despite these limitations, our study provides valuable data on the prevalence, reasons, and timing for WLST decisions in OHCA patients with ECPR using a large nationwide cohort. Further research is needed to investigate more accurate neurologic prognostication in the early phase in OHCA/ECPR patients to allow more in-depth WLST decision pathways, which could guide clinicians in making medically and ethically appropriate decisions.

## Conclusions

WLST decisions were made for about one-third of ECPR/OHCA patients in a Japanese nationwide cohort. The main reason for WLST decisions was perceived unfavorable neurological prognosis. Decisions were made early, at a median of two days after ICU admission during a time when neurological prognostication is not recommended. Decisions and neurological assessments for ECPR/OHCA patients need further analysis.

## Supplementary Information

**Additional file 1.** The days of WLST decisions are shown with each WLST decision reason. The trend for WLST did not differ by WLST reason. WLST: withholding/withdrawal of life-sustaining therapy

**Additional file 2.** Kaplan–Meier survival curve was drawn for the comparison of 30-day survival of withholding of life-sustaining therapy patients and withdrawal of life-sustaining therapy patients with log-rank test. Thirty-day survival was better in the withholding of life-sustaining therapy patients (*p *< 0.001)

## Data Availability

The datasets used and/or analyzed during the current study are available from the authors upon reasonable request.

## Abbreviations

CPC: Cerebral Performance Category
ECMO: Extracorporeal membrane oxygenation
ECPR: Extracorporeal cardiopulmonary resuscitation
ICU: Intensive care unit
OHCA: Out-of-hospital cardiac arrest
OR: Odds ratio
PS: Performance status
ROSC: Return of spontaneous circulation
SOFA: Sequential organ failure assessment
WLST: Withdrawal/withholding of life-sustaining therapy
