# Supplementary figures and images for: Prevalence, reasons, and timing of decisions to withhold/withdraw life-sustaining therapy for out-of-hospital cardiac arrest patients with extracorporeal cardiopulmonary resuscitation
Source: Crit Care. 2023 Jun 27;27:252. doi: 10.1186/s13054-023-04534-2 (PMC10294328; doi:10.1186/s13054-023-04534-2)

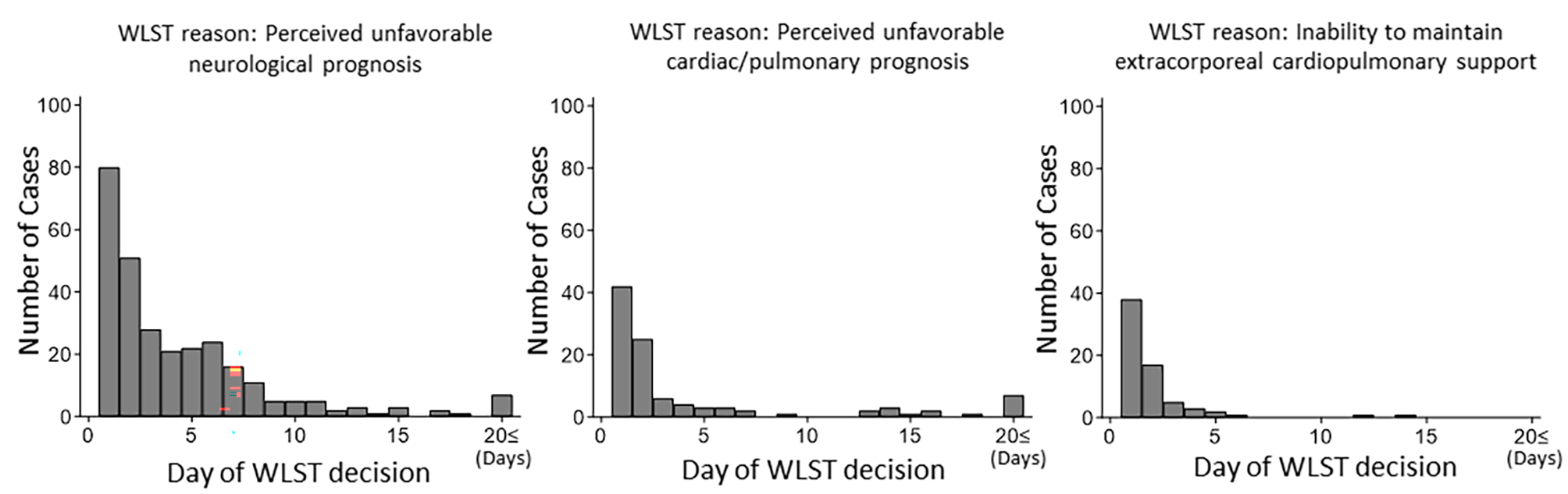

Supplement: Supplementary file 1 — Additional file 1. The days of WLST decisions are shown with each WLST decision reason. The trend for WLST did not differ by WLST reason. WLST: withholding/withdrawal of life-sustaining therapy [file 13054_2023_4534_MOESM1_ESM.tif]

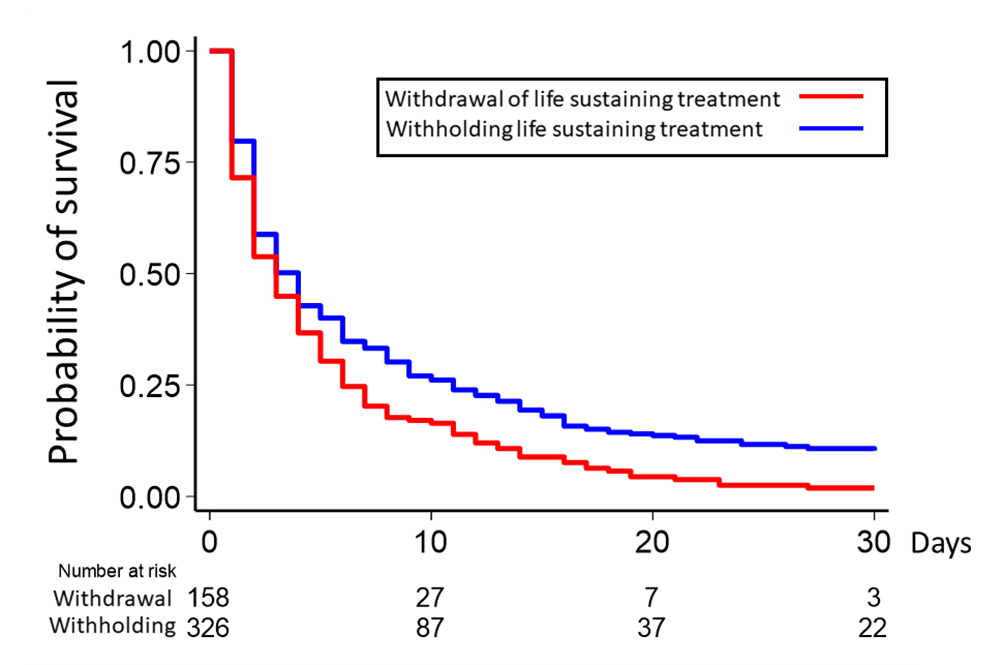

Supplement: Supplementary file 2 — Additional file 2. Kaplan–Meier survival curve was drawn for the comparison of 30-day survival of withholding of life-sustaining therapy patients and withdrawal of life-sustaining therapy patients with log-rank test. Thirty-day survival was better in the withholding of life-sustaining therapy patients (p < 0.001) [file 13054_2023_4534_MOESM2_ESM.tif]
